# Optical redox imaging to screen synthetic hydrogels for stem cell-derived cardiomyocyte differentiation and maturation

**DOI:** 10.1117/1.BIOS.1.1.015002

**Published:** 2024-05-20

**Authors:** Danielle E. Desa, Margot J. Amitrano, William L. Murphy, Melissa C. Skala

**Affiliations:** a Morgridge Institute for Research, Madison, Wisconsin, United States; b University of Wisconsin-Madison, Department of Biomedical Engineering, Madison, Wisconsin, United States; c University of Wisconsin-Madison, Department of Orthopedics and Rehabilitation, Madison, Wisconsin, United States

**Keywords:** label-free imaging, hydrogel, cardiomyocyte, cell manufacturing, induced pluripotent stem cell, autofluorescence

## Abstract

**Significance:**

Heart disease is the leading cause of death in the United States, yet research is limited by the inability to culture primary cardiac cells. Cardiomyocytes (CMs) derived from human induced pluripotent stem cells (iPSCs) are a promising solution for drug screening and disease modeling.

**Aim:**

Induced pluripotent stem cell-derived CM (iPSC-CM) differentiation and maturation studies typically use heterogeneous substrates for growth and destructive verification methods. Reproducible, tunable substrates and touch-free monitoring are needed to identify ideal conditions to produce homogenous, functional CMs.

**Approach:**

We generated synthetic polyethylene glycol-based hydrogels for iPSC-CM differentiation and maturation. Peptide concentrations, combinations, and gel stiffness were tuned independently. Label-free optical redox imaging (ORI) was performed on a widefield microscope in a 96-well screen of gel formulations. We performed live-cell imaging throughout differentiation and early to late maturation to identify key metabolic shifts.

**Results:**

Label-free ORI confirmed the expected metabolic shifts toward oxidative phosphorylation throughout the differentiation and maturation processes of iPSC-CMs on synthetic hydrogels. Furthermore, ORI distinguished high and low differentiation efficiency cell batches in the cardiac progenitor stage.

**Conclusions:**

We established a workflow for medium throughput screening of synthetic hydrogel conditions with the ability to perform repeated live-cell measurements and confirm expected metabolic shifts. These methods have implications for reproducible iPSC-CM generation in biomanufacturing.

Statement of Discovery We demonstrate widefield redox imaging as a tool for monitoring cardiomyocyte development on novel hydrogels.

## 1 Introduction

Cardiovascular disease remains the leading cause of death in the United States^1^ and costs the healthcare system over $200 billion annually.^2^ Heart diseases remain difficult to treat and study both due to the lack of regenerative potential in the human heart and the inability to maintain primary cardiomyocytes (CMs; contractile heart muscle cells) in culture. Human induced pluripotent stem cell-derived CMs (iPSC-CMs) are of great interest to the biomanufacturing industry, with promising applications that include improved drug screening platforms, accurate disease models, and personalized regenerative medicine. iPSCs can be effectively reprogrammed to generate high-purity CM batches.^3^^,^^4^ However, *in vitro* maturation of iPSC-CMs remains difficult, with cells demonstrating a fetal-like phenotype characterized by disorganized structure and decreased electrophysiological capabilities.^5^^,^^6^ Stem cell-derived CMs also exhibit batch-to-batch and line-to-line variability in function and structure.^7^^–^^11^ Furthermore, methods to assess differentiation and maturation efficiency [e.g., ribonucleic acid (RNA) sequencing, immunostaining] are generally destructive and low-throughput.^12^^,^^13^ The efficient production and widespread use of iPSC-CMs will require consistent manufacturing of functionally mature cell batches and touch-free, higher throughput, and nondestructive monitoring.

Various techniques have been employed to improve all facets (e.g., bioenergetic, structural, electromechanical) of iPSC-CM maturation; these include electromechanical stimulation, extended culture, media additives, and co-culture with other cell types.^14^^,^^15^ Culture substrate is one critical regulator of cell behavior *in vitro*. For example, the composition (e.g., extracellular matrix proteins, synthetic polymers) and stiffness of the substrate affect the motility^16^^–^^18^ and morphology^18^^–^^20^ of many cell types. Substrate properties also impact stem cell differentiation and maturation potential,^20^^–^^24^ and synthetic hydrogels with tunable physical, mechanical, and chemical properties^25^^,^^26^ present a method for regulating stem cell fate in culture. Synthetic substrates show promise for iPSC culture and targeted differentiations^14^^,^^25^^,^^27^^,^^28^ although improvements are needed in terms of maturation. iPSC-CM culture has traditionally relied on Matrigel, a mixture of basement membrane proteins derived from murine sarcomas. However, the composition, mechanical properties, and potential for xenogenic contamination vary among Matrigel batches, and it cannot be manipulated to fine-tune substrate properties and control degradation.^25^^,^^27^^,^^29^ Synthetic substrates, therefore, may be an attractive alternative for robust iPSC-CM manufacturing.

Verifying successful iPSC-CM differentiation and/or maturation on any substrate typically relies on techniques such as quantitative polymerase chain reaction (qPCR), immunostaining, and flow cytometry for cardiac markers.^3^^,^^12^^,^^13^ While highly specific, these methods are destructive and can therefore only be performed at a single timepoint. Label-free optical imaging and sensing techniques are attractive touch-free solutions for repeated monitoring during cellular development.^30^ Different modalities can detect the metabolic, organizational, and structural changes that occur during stem cell differentiation and subsequent maturation of the resulting cell types. Optical redox imaging (ORI) makes use of the intrinsic fluorescent properties of reduced nicotinamide adenine dinucleotide (phosphate) [NAD(P)H] and oxidized flavin adenine dinucleotide (FAD), two coenzymes involved in hundreds of cellular metabolic reactions.^31^^–^^34^ Multiphoton imaging of NAD(P)H and FAD fluorescence intensities has been used to quantify changes that occur during mesenchymal stem cell differentiation into chondrocytes,^35^ osteocytes,^35^^,^^36^ and adipocytes.^37^^–^^39^ In addition, the fluorescence lifetimes of NAD(P)H and FAD are distinct in their protein-bound and free states and provide information on environmental factors (e.g., pH, oxygen).^33^^,^^34^^,^^40^^,^^41^ Label-free fluorescence lifetime imaging microscopy (FLIM) has been used to distinguish undifferentiated stem cells and progenitors from their differentiated counterparts,^42^^–^^45^ separate high and low differentiation efficiency groups,^46^ and perform long-term monitoring during maturation.^47^^,^^48^ Other label-free imaging modalities can monitor structural changes that occur during stem cell differentiation.^49^^–^^51^ Importantly, these techniques are repeatable and live cell-compatible, allowing traditional endpoint analyses to be performed on the same cell batches.

iPSCs primarily use glycolysis for adenosine triphosphate (ATP) production and shift to oxidative phosphorylation during differentiation to CMs,^52^^,^^53^ following the metabolic trends of fetal and adult CMs, respectively.^54^ Here, we used label-free optical imaging of NAD(P)H and FAD to verify these expected metabolic changes in cells developing on synthetic hydrogels. Polyethylene glycol (PEG)-based hydrogels were crosslinked with fibronectin and laminin-derived peptides and tuned to relevant physiological stiffnesses. We used autofluorescence FLIM to verify increased oxidative metabolism and NAD(P)H protein binding activity throughout differentiation. To increase throughput and screen more hydrogel formulations, we next used widefield NAD(P)H and FAD autofluorescence imaging to calculate the optical redox ratio (ORR, defined as the fluorescence intensity ratio of NAD(P)H/(NAD(P)H+FAD) at multiple timepoints throughout differentiation. We found that ORR decreased in cells cultured on all synthetic gel formulations during differentiation, early maturation (days 16 to 30) and late maturation (day 30+), indicating a decline in glycolytic metabolism and an increase in oxidative phosphorylation. Finally, we observed metabolic differences in high and low differentiation efficiency cells, with groups exhibiting significantly different ORR in the cardiac progenitor stage. These results suggest that widefield autofluorescence ORI can be used repeatedly during iPSC-CM differentiation and maturation on synthetic hydrogels to monitor cell metabolism and may be a useful screening tool for cell manufacturing.

## 2 Methods

### 2.1 Synthetic Hydrogels

Hydrogels were prepared using 8-arm 20 kDa norbornene-functionalized PEG (JenKem Technology, Plano, Texas, United States), 3.4 kDa PEG di-thiol (Laysan Bio, Arab, Alabama, United States) crosslinker, 0.1  wt/wt% Irgacure 2959 (CIBA, Basel, Switzerland) photoinitiator, diluted with phosphate-buffered saline (PBS) to reach desired concentrations. The following peptides were added at varying concentrations: CRGDSP (referred to as L), head-to-tail cyclized RGDfC (cRGDgF, or C), CIKVAV (I), and CYIGSR (Y) (Table S1 in the Supplementary Material). All peptides were purchased from GenScript. Here, we developed hydrogels demonstrating physiologically relevant stiffnesses, corresponding to neonatal (0.5 and 2 kPa), healthy adult (4 and 6 kPa), and fibrotic adult (10 kPa) cardiac tissue.^55^ Hydrogel formulations are written as peptide concentration in mM, single-letter peptide abbreviation, and stiffness in kPa (e.g., 7 mM cRGDfC 3 mM CIKVAV 2 kPa is written 7C-3I 2). Hydrogel precursor solutions were exposed to ultraviolet light (UV, 365 nm, $4.5\;{\mathrm{mW}/\mathrm{cm}}^{2}$, 5 min) for polymerization. Hydrogels were then incubated in RPMI 1640 medium (Gibco) at 37°C until use. All gel formations for a particular experiment (summarized in Tables S1–S6 in the Supplementary Material) were made in triplicate.

Matrigel (Corning, Corning, New York, United States) was used as a control substrate in three forms: Matrigel diluted in DMEM/F12 (ThermoFisher Scientific, Waltham, Massachusetts, United States) medium forming a thin plate coating (referred to as “coated plates” throughout the text), undiluted Matrigel forming a gel (“Matrigel”), and undiluted growth factor reduced “Geltrex” forming a gel (ThermoFisher Scientific). The coated plate represents a widely used standard culture substrate for iPSC-CMs.^3^

### 2.2 iPSC Maintenance

Human iPSCs (WTC-11) were maintained on coated plates and fed daily with mTeSR1 medium (StemCell Technologies, Vancouver, Canada). Cells were passaged every 3 to 4 days using Versene (ThermoFisher Scientific) and replated onto freshly coated plates in mTeSR1 supplemented with 5  μM Y-27632 (Tocris, Bristol, United Kingdom).

### 2.3 CM Differentiation

CM differentiation was performed as previously described.^3^ Briefly, iPSCs were passaged using Accutase (ThermoFisher Scientific) and plated on fresh coated plates in mTeSR1 supplemented with 5  μM Y-27632 at a cell density of $40,000\;{\text{cells}/\mathrm{cm}}^{2}$. iPSCs were maintained for 2 days prior to differentiating. CM differentiation was initiated (day 0) by activating Wnt signaling with 7  μM CHIR99021 (Selleckchem, Houston, Texas, United States) in RPMI 1640 (Gibco, Billings, Montana, United States) and B27 supplement without insulin (LifeTechnologies, Carlsbad, California, United States). Wnt signaling was inhibited with 5  μM IWP2 (Tocris) in RPMI/B27 without insulin after 48 h (day 2). Cells were maintained in RPMI/B27 plus insulin (LifeTechnologies) beginning day 4.

On day 5 of differentiation, cardiac progenitor cells (CPCs) were lifted and singularized using Accutase, frozen in 60% RPMI/B27 without insulin, 30% fetal bovine serum, and 10% dimethyl sulfoxide (DMSO), and stored in liquid nitrogen. Cells were thawed as needed and replated in RPMI/B27 without insulin with 5  μM Y-27632 at a 1:1 seeding ratio on Matrigel, Geltrex, or synthetic hydrogels in a 96-well angiogenesis plate (Ibidi, Fitchburg, Wisconsin, United States). The media was switched to RPMI/B27 plus insulin after 24 h, and cells were fed every 48 h until day 10 and every 72 h after.

### 2.4 Flow Cytometry

Cells were assessed for differentiation efficiency by cardiac troponin (cTnT) expression on day 16 and on day 30 for expression of the early maturation marker myosin light chain-2 (MLC2). Flow cytometry was performed as previously described.^3^ Briefly, cells were dissociated with Accutase (ThermoFisher Scientific), fixed in 1% paraformaldehyde for 20 min at room temperature, and incubated in 90% methanol for 20 min at 4°C before storing at −20°C until staining. Cells were washed twice in flow buffer 1 (FB-1) consisting of PBS and 0.5% bovine serum albumin (ThermoFisher Scientific) and then incubated in flow buffer 2 (FB-2) consisting of FB-1 and 1% Triton-X 100 (ThermoFisher Scientific) and the primary antibody at 4°C overnight. Samples were washed with FB-2 and incubated with a secondary antibody in FB-2 at room temperature for 30 min in the dark. Cells were washed in FB-2 and resuspended in FB-1 for processing. Flow cytometry was performed on an Attune NxT (ThermoFisher Scientific), and data were analyzed using FCS Express. Primary antibodies used were cTnT (Abcam, Cambridge, United Kingdom, 1:800), MLC2a (Synaptic Systems, Göttingen, Germany, 1:200), and MLC2v (ProteinTech, Rosemont, Illinois, United States, 1:200). The secondary antibodies used were AlexaFluor 488 and 568 (Abcam, 1:1000).

### 2.5 Fluorescence Lifetime Imaging and Analysis

Multiphoton imaging was performed using a custom-built multiphoton microscope (Ultima, Bruker, Madison, Wisconsin, United States) consisting of an inverted microscope body (Ti-E, Nikon, Tokyo, Japan) coupled to an ultrafast tunable laser source (80 MHz, 100 fs pulses; Insight DS+, Spectra Physics, Santa Clara, California, United States). Images were acquired using time-correlated single-photon counting electronics (SPC 150, Becker & Hickl GmbH, Berlin, Germany) using Prairie View software (Bruker). NAD(P)H was excited at 750 nm (~8 mW at the sample) using a 20× water immersion, 1.0 NA objective (Plan Apo, Zeiss, Jena, Germany) with 1× optical zoom, 4.8  μs pixel dwell time, 60-s integration time, and image size of 512×512  pixels. NAD(P)H emissions were separated from excitation light using a 720 long pass filter and collected using a GaAsP photomultiplier tube (H7422, Hamamatsu, Shizuoka, Japan) and a 466/40  nm bandpass filter. A single field of view (FOV, ∼300  μm×300  μm) was taken of each gel. Images were acquired on days 6, 8, 10, and 16 post-differentiation. The instrument response function was measured using a second-harmonic generation signal from urea crystals excited at 890 nm, with full width at half-maximum of 240 ps.

Fluorescence lifetime components were computed for each image pixel using SPCImage (v8.0, Becker & Hickl GmbH). The background was thresholded, and the pixel-wise decay curves were fit to a biexponential model convolved with the instrument response function, using an iterative parameter optimization to obtain the lowest sum of the squared differences between the model and data (weighted least squares algorithm). The two-component model is $I(t)=\alpha_{1}e^{-t/\tau_{1}}+\alpha_{2}e^{-t/\tau_{2}}+C$, where I(t) is the fluorescence intensity at time t after the laser excitation pulse, $\tau_{1}$ and $\tau_{2}$ are the fluorescence lifetimes of the short and long lifetime components, respectively, $\alpha_{1}$ and $\alpha_{2}$ are the fractional contributions of the short and long lifetime components, respectively, and C accounts for background.^33^^,^^56^ The mean fluorescence lifetime is defined as $\tau_{m}=\alpha_{1}\tau_{1}+\alpha_{2}\tau_{2}$. To enhance the fluorescence counts in each decay, a 3×3 bin comprising nine neighboring pixels was employed (>1000  photons/pixel^57^). Whole-cell masks were generated using Cellpose (model “cyto2”) and manually corrected as needed.^58^^,^^59^ Masks were binarized, and FLIM data were extracted from all cells in a single FOV using a custom Python library, “cell analysis tools.”^60^ Plots and statistics were generated using GraphPad Prism 10.

### 2.6 Widefield ORI and Analysis

Widefield ORI was performed using a Nikon Ti-2E coupled to a SOLA light engine (380 to 660 nm, Lumencor, Beaverton, Oregon, United States). All data were collected with NIS Elements software (Nikon) using a 10× air, 0.45 NA objective (Plan Apo, Nikon), and Hamamatsu Orca-Flash digital CMOS camera. NAD(P)H was excited for 300 to 500 ms through a 360/40  nm filter at 50% power ($0.0011\;{\mathrm{mW}/\mathrm{mm}}^{2}$), and emissions were collected using a 400 nm dichroic mirror and a standard 4′,6-diamidino-2-phenylindole (DAPI, 460/50  nm) filter. FAD was sequentially excited for 1.3 to 1.7 s through a 480/30 nm filter at 50% power ($0.0019\;\mathrm{mW}/{\mathrm{mm}}^{2}$) and emissions collected using a 505 nm dichroic mirror and a standard fluorescein isothiocyanate (FITC, 535/20 nm) filter. A single FOV (image size=1.33  mm×1.28  mm,2044×2048  pixels) was taken of each gel, with three replicates per gel formulation. Images were acquired on days 6, 8, 10, and 16 post-differentiation (differentiation); on days 6, 8, 10, and every 3 days thereafter through day 30 (early maturation); or on days 6, 8, 10, 16, 23, 30, and every 10 days thereafter (late maturation).

**Fig. 1:**
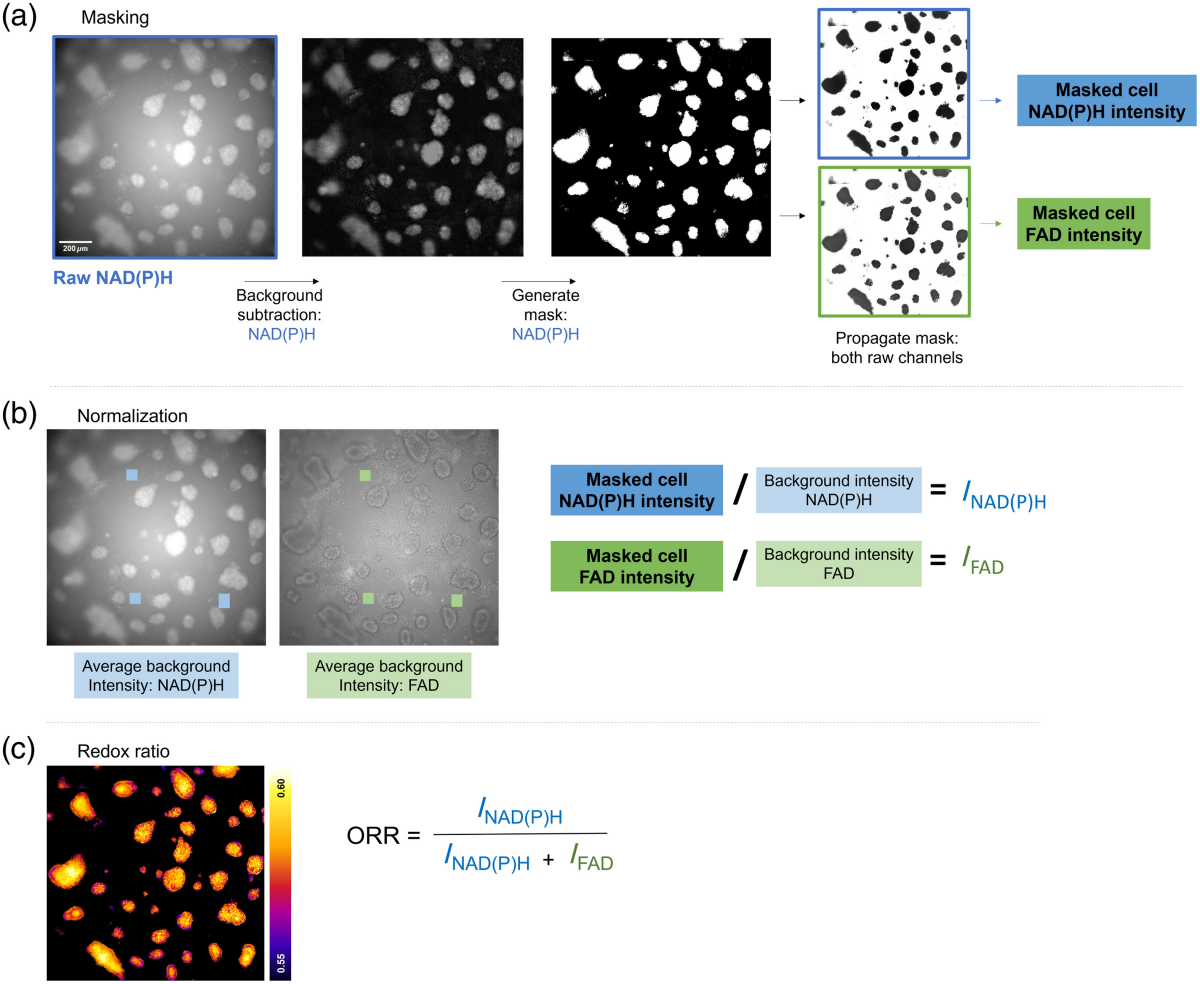
Widefield optical redox analysis workflow. (a) A rolling ball subtraction was performed on raw NAD(P)H images prior to thresholding, generating binary masks separating cell autofluorescence from the background. This mask was applied to both raw image channels to measure cellular NAD(P)H and FAD in each FOV. (b) The average intensity of three cell-free areas was measured to account for gel autofluorescence. Cellular NAD(P)H and FAD signal were normalized to background intensity on a per-gel level. (c) Background-normalized NAD(P)H and FAD intensity values were used to calculate ORR per FOV. Scale bar=200  μm.

NAD(P)H and FAD widefield autofluorescence images were analyzed using Fiji.^61^ A rolling ball subtraction was performed to correct vignetting on the NAD(P)H fluorescence intensity image. The resulting image was thresholded to separate cells from the background signal (e.g., substrate autofluorescence) and binarized to create a mask, where background pixels are assigned a value of 0 and pixels within cells a value of 1 (Fig. 1). This mask was multiplied by both the raw NAD(P)H and FAD intensity images, and the mean fluorescence intensity of the masked cell pixels was measured from each channel. We expect day-to-day variation in the widefield system (e.g., minor drift, variation in exposure time), and illumination through an autofluorescent substrate may affect intensity-based measurements. To account for these factors, we measured the average fluorescence intensity from three background (cell-free) locations within each gel. We calculated a background-normalized redox ratio for all cells on each gel as follows (summarized in Fig. 1):

INAD(P)H=Icell NAD(P)H/Ibackground NAD(P)H,

IFAD=Icell FADIbackground FAD,

$$\mathrm{Normalized}\;\mathrm{ORR}=\frac{I_{\mathrm{NA}(P)H}}{I_{\mathrm{NAD}(P)H}+I_{\mathrm{FAD}}}.$$

All plots and statistics were generated using GraphPad Prism 10.

## 3 Results

### 3.1 NAD(P)H Protein Binding Increases during iPSC-CM Differentiation on Synthetic Hydrogels

**Fig. 2:**
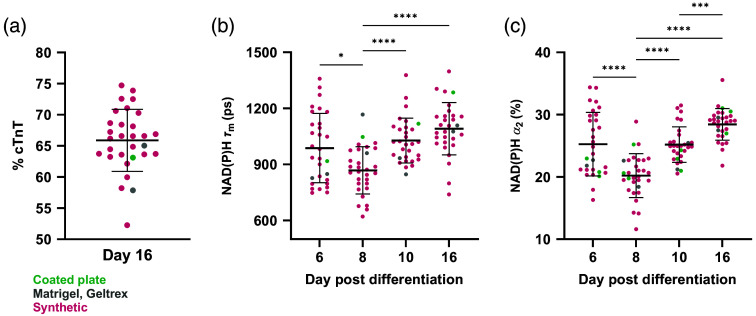
NAD(P)H protein binding increases during iPSC-CM differentiation on synthetic hydrogels. CPCs were seeded on synthetic hydrogels (pink, 27 formulations), coated plates (green), and Matrigel or Geltrex (gray) with three gel replicates per condition. (a) Differentiation was confirmed using flow cytometry for cTnT expression (% cTnT). (b) Average NAD(P)H lifetime ($\tau_{m}$) and (c) protein-bound fraction ($\alpha_{2}$) were measured per gel and shown to increase throughout differentiation, indicating more oxidative metabolism. Repeated measures analysis of variance (ANOVA) with Tukey’s multiple comparisons test, *p<0.05, ***p<0.001, and ****p<0.0001. Each dot=cells from three replicate gels.

CMs undergo extensive metabolic changes during development, exhibiting an early reliance on glycolysis and shifting primarily to fatty acid oxidation during terminal differentiation.^54^ Upregulation of oxidative phosphorylation increases NADH protein binding, which can be detected noninvasively using autofluorescence FLIM.^33^^,^^40^ To date, autofluorescence FLIM has not been used to study metabolic changes in iPSC-CMs throughout the full differentiation and >30  day maturation processes. Furthermore, repeated label-free metabolic imaging has not been performed on iPSC-CMs undergoing these processes on synthetic substrates. Multiphoton NAD(P)H FLIM was therefore first used to characterize expected metabolic shifts and complement later widefield redox analysis. Imaging was performed at the CPC stage (day 6) through differentiation (day 16). Twenty-seven synthetic hydrogel formations and coated plates, Matrigel, and Geltrex were seeded from the same batch of CPCs (Table S2 in the Supplementary Material). The imaged cells were fixed on day 16, and flow cytometry was performed to verify differentiation efficiency [Fig. 2(a)]. Most cells exhibited high (>60%) levels of cTnT expression, regardless of substrate. We observed an increase in NAD(P)H mean lifetime ($\tau_{m}$) and the protein-bound fraction ($\alpha_{2}$) by day 10 relative to days 6 and 8, with further increases by day 16 [Figs. 2(b), 2(c)]. This indicates that cellular metabolism shifts to a more oxidative phenotype during differentiation and confirms the expected changes in cells differentiated on synthetic substrates.

### 3.2 Widefield ORI Maintains Viability and Purity of iPSC-CMs

**Fig. 3:**
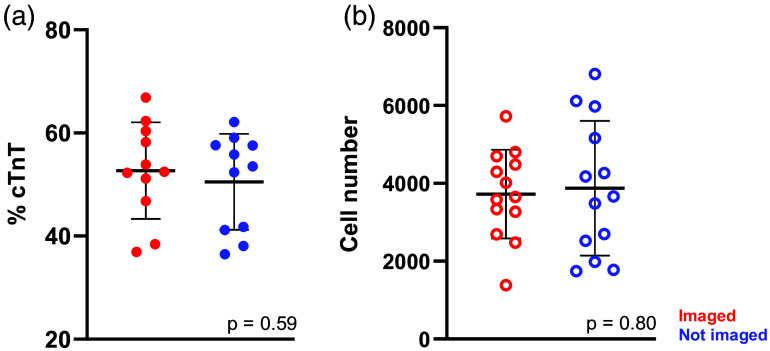
Widefield redox imaging with UV excitation is minimally phototoxic to iPSC-CMs. Twelve representative gel formulations were seeded with CPCs and imaged on days 6, 8, 10, and 16 post-differentiation. A matched set of cells in the same plate were not imaged. (a) Flow cytometry for differentiation markers (%cTnT) and (b) cell number on day 16 revealed no significant differences between the imaged and non-imaged groups, indicating minimal phototoxic effects of ORI during differentiation. Unpaired t-test. Each dot=cells from three replicate gels.

Multiphoton NAD(P)H FLIM is sensitive to the local molecular environment and can achieve high spatial resolution. However, acquiring and analyzing multiphoton FLIM images are time-consuming, requiring expensive hardware and specialized training. As a result, we next tested whether single-photon ORI with a commercial epifluorescent widefield microscope provides similar sensitivity to screen iPSC-CM differentiation and maturation conditions. First, to ensure UV light exposure did not impact differentiation, CPCs were plated on 12 representative gel formulations (plate coating, Matrigel, Geltrex, 9 synthetics, Table S3 in the Supplementary Material) each with three replicates (36 gels total). Two experimental gel sets were generated in the same 96-well angiogenesis plate, and one set was imaged on days 6, 8, 10, and 16 using ORI. The other set was subjected to the same environmental and media changes (e.g., moving between incubators) but not directly exposed to UV light. Flow cytometry was performed on day 16 to assess differentiation outcome (% cTnT and cell number). No significant differences (p>0.05) were observed between the UV-exposed and non-exposed groups (Fig. 3).

### 3.3 Widefield ORR Decreases during iPSC-CM Differentiation on Synthetic Hydrogels

**Fig. 4:**
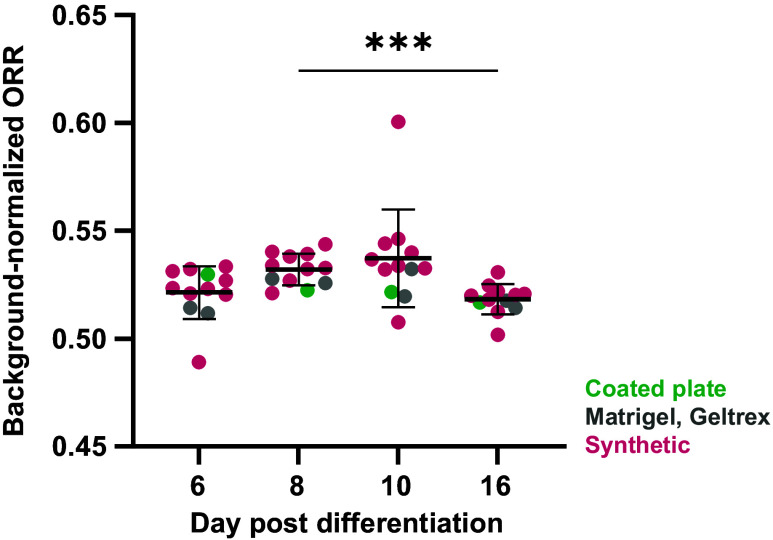
Widefield ORR shifts during iPSC-CM differentiation on synthetic hydrogels. CPCs were seeded on synthetic hydrogels (nine formulations), coated plates, Matrigel, or Geltrex, with three gel replicates per screen. ORR initially increases and then begins to decrease by the differentiation timepoint, indicative of decreasing glycolytic activity. Repeated measures ANOVA with Tukey’s multiple comparisons test, ***p<0.001. Each dot=cells from nine replicate gels (three per screen).

We next investigated if the expected metabolic shift away from glycolysis could be observed using widefield ORI performed using a commercial microscope and free analysis software. Twelve gel conditions were generated in triplicate (Table S3 in the Supplementary Material), and CPCs were seeded 5 days post-differentiation. ORR was calculated from widefield fluorescence intensity images on days 6, 8, 10, and 16 post-differentiation before cells were fixed for flow cytometry in three separate screens (the average for all three screens was ≥50% cTnT for each gel type). ORR initially increases in the CPC stage (6 to 10) and then decreases by the differentiation timepoint (day 16, Fig. 4).

### 3.4 Widefield ORR Can Monitor iPSC-CM Metabolic Maturation on Synthetic Hydrogels

**Fig. 5:**
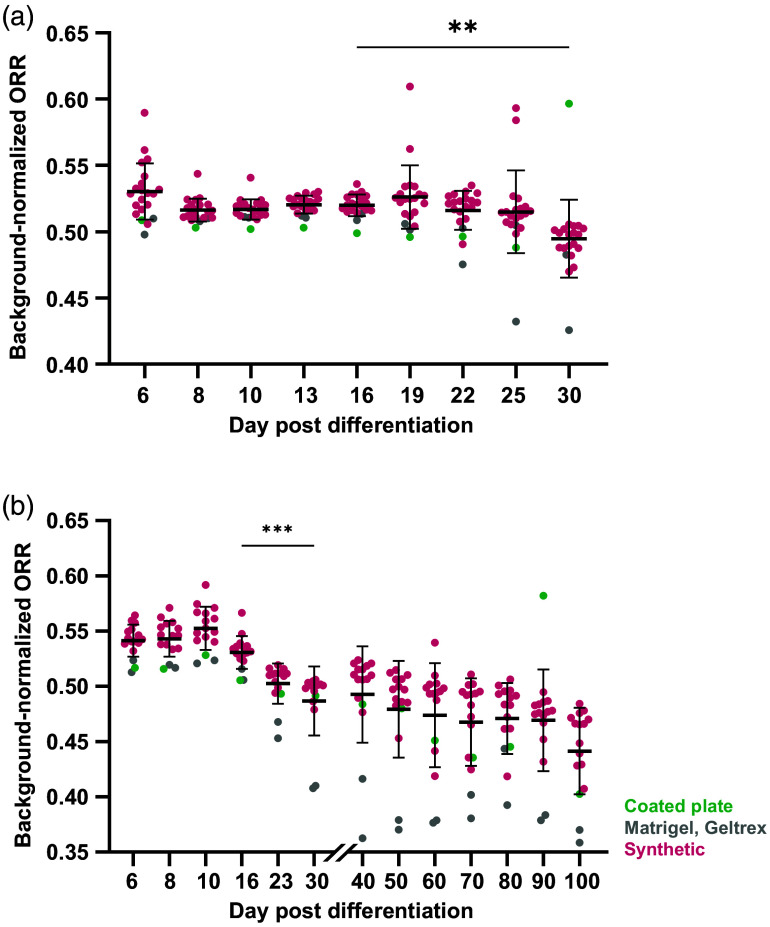
Widefield ORR becomes more oxidized during early and late iPSC-CM maturation. CPCs were seeded on synthetic hydrogels, coated plates, Matrigel, or Geltrex with three gel replicates per formulation. (a) ORR was measured at multiple timepoints through early (up to day 30) or late maturation (day 30+). ORR significantly decreased relative to ORR at the differentiation timepoint (day 16 versus 30). (b) ORR continued declining during late maturation (days 30 to 100), with cells on synthetic hydrogels showing further decreases out to day 100. Brown–Forsythe and Welch ANOVA with Dunnett’s T3 multiple comparisons test, **p<0.01, ***p<0.001. Each dot=cells from three replicate gels.

We next investigated if cell metabolism could be monitored in maturing iPSC-CMs using widefield ORI. Eighteen different synthetic gel formulations plus plate coatings, Matrigel, and Geltrex (Table S4 in the Supplementary Material) were made in triplicate and seeded with high differentiation efficiency cells (>70% of cells become CMs in prior experiments from the same batch, confirmed by flow cytometry).^3^ ORR was measured at days 6, 8, and 10 and then every 3 days thereafter until early maturation (day 30). We found that ORR calculated from widefield fluorescence images was sensitive to metabolic changes occurring post-differentiation, and by day 30, ORR of cells seeded on all gel formulations significantly decreased relative to day 16 [Fig. 5(a)].

Long-term culture (beyond early maturation) is known to enhance iPSC-CM maturation, resulting in increased cell size, alignment, and maturation-related gene and protein expression.^15^^,^^62^ We performed extended culture and widefield imaging up to 100 days post-differentiation to observe if ORI is sensitive to further metabolic changes that occur across this timescale. We found that iPSC-CM ORR continued to decrease beyond the early maturation timepoint (day 30) and reached its lowest measured point at 100 days, indicating further increases in oxidative metabolism throughout extended culture [Fig. 5(b)]. This trend was most notable in the synthetic hydrogels, with significant decreases observed between the early (day 30) and late (days 90 to 100) stages (Fig. S1 in the Supplementary Material).

### 3.5 Widefield ORR Distinguishes Between High and Low Differentiation Efficiency of Batches in the Cardiac Progenitor Stage

Confirming batch purity of stem cell-derived CMs is often labor-intensive, requiring techniques such as flow cytometry or qPCR that are typically performed two weeks after initializing differentiation.^3^^,^^8^^,^^9^^,^^63^ Label-free FLIM of metabolic coenzymes can separate stem cell-derived CMs by differentiation efficiency within 24 hours of initializing differentiation.^46^ We investigated if widefield ORI provides sufficient sensitivity to separate cell batches by differentiation efficiency in the CPC stage. Seventeen gel formulations (Table S6 in the Supplementary Material) were generated in triplicate and duplicated in a second plate. One plate was seeded with high-efficiency cells (>70% CM population expected) and the second with low-efficiency cells (<35% CM population expected). Plates were imaged in the same session on days 6, 8, and 10 and then every 3 days thereafter until day 30. Cells were fixed on day 30, and flow cytometry for an early structural marker of CM maturation (MLC2)^64^^,^^65^ was performed as an independent measure of maturity.

**Fig. 6:**
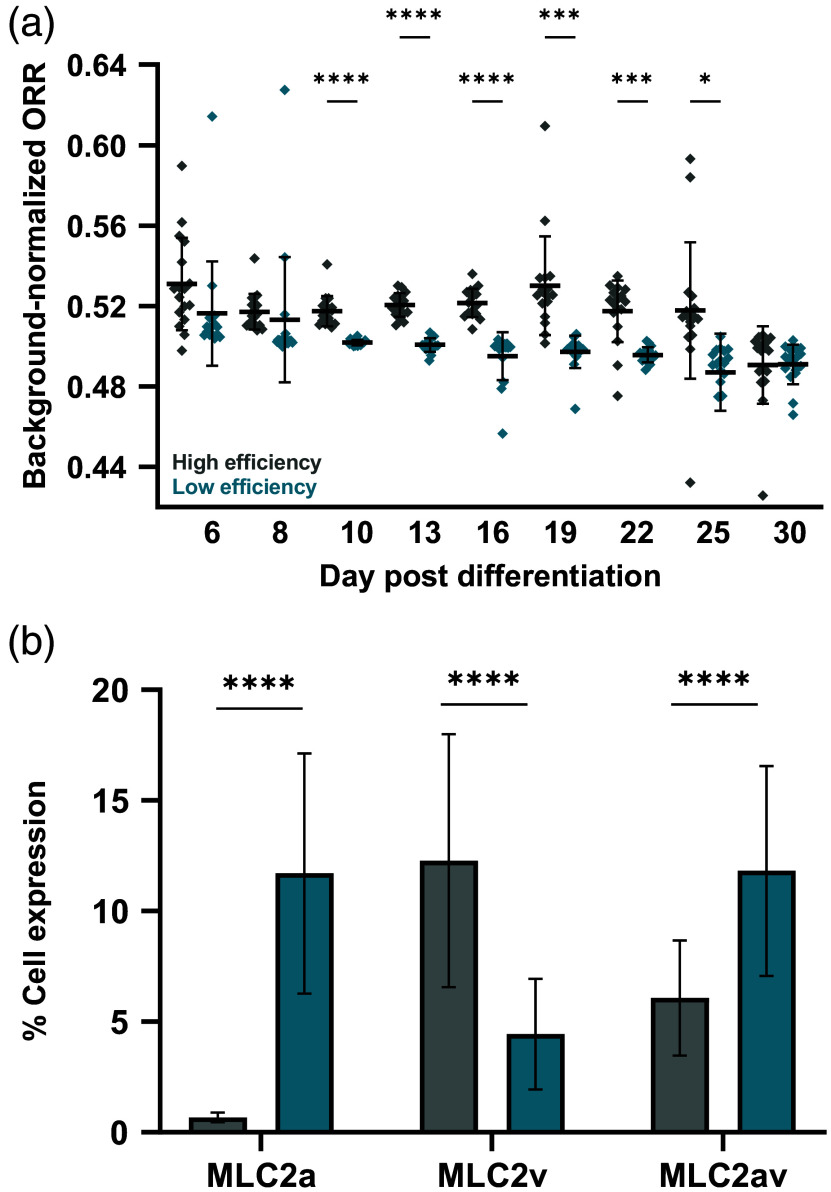
Widefield ORI separates high and low differentiation efficiency groups at the cardiac progenitor stage. High- and low-efficiency CPCs were seeded on identical gels, and widefield ORI was measured through early maturation. (a) ORR significantly differs between efficiency groups at the CPC stage, with high-efficiency cells initially having a higher ORR. This distinction disappears by early maturation (day 30). High-efficiency cells exhibit a lower ORR and more oxidative phenotype by day 30 relative to day 6. (b) High-efficiency cells express greater levels of the ventricular isoform of MLC2, indicating increased structural maturity relative to their low-efficiency counterparts. Welch’s t-test, ****p<0.0001. Brown–Forsythe and Welch ANOVA with Dunnett’s T3 multiple comparisons test, *p<0.05, ***p<0.001, and ****p<0.0001. Each dot=cells from three replicate gels.

In the early CPC stage (day 6), low-efficiency cells had a significantly lower ORR compared with high-efficiency cells, and this difference persisted beyond differentiation [day 16, Fig. 6(a)]. As cells began to mature, the distinction between the groups was lost (day 30). High-efficiency cells exhibited greater decreases in ORR throughout maturation (−8.15% average ORR day 6 versus 30) compared with low-efficiency cells [−5.09% average ORR day 6 versus 30, Fig. 6(a)]. On day 30, the higher-efficiency cells also exhibited higher expression levels of the ventricular isoform of the myosin light chain [MLC2v, Fig. 6(b)], one structural indicator of CM maturity, whereas the low-efficiency cells expressed more of the atrial and intermediate forms [MLC2a, av, Fig. 6(b)]. These data support a more mature phenotype in the high-efficiency cells on day 30, with lower ORR indicating a shift toward oxidative metabolism and greater metabolic maturity and higher MLC2v expression indicating more structural maturity.

## 4 Discussion

Here, we report a widefield optical imaging method to monitor metabolic changes in iPSC differentiation and subsequent CM maturation. We used NAD(P)H multiphoton FLIM to confirm an oxidative shift in iPSCs undergoing differentiation on PEG-based hydrogels. We used widefield ORI to decrease imaging time and complexity and observed a decrease in ORR, indicating more oxidative metabolism, with differentiation and maturation for cells on multiple hydrogel formulations. We demonstrated extended culture on synthetic hydrogels and repeatedly performed widefield ORI up to 100 days post-differentiation and observed redox changes throughout maturation. Finally, widefield ORI distinguished differentiation efficiencies between CPCs as early as 6 days post-differentiation, with higher-efficiency cells exhibiting both a greater decrease in ORR and increased structural maturity (%MLC2v) by day 30 compared with lower-efficiency cells.

To date, label-free metabolic imaging has not been performed in iPSC-CMs differentiating and/or maturing on synthetic substrates. Therefore, both widefield ORI and NAD(P)H FLIM were performed to provide a comprehensive and complementary characterization of autofluorescence in these samples, which have not been previously imaged using autofluorescence FLIM or intensity methods. For the imaging parameters used in this study, widefield ORI reduces imaging and analysis time approximately fourfold compared with multiphoton autofluorescence FLIM. Multiphoton FLIM provides subcellular resolution, optical sectioning, and is sensitive to changes in the molecular state and environment. While both FLIM and widefield redox imaging are both sensitive to redox pool changes, they have differing specificities.^34^ As a result, we do not necessarily expect a one-to-one correspondence between our NAD(P)H FLIM and ORI data.^34^^,^^40^^,^^66^^–^^68^ However, a widefield imaging configuration presents certain advantages such as lower cost, wide availability, and suitability for rapid screening of multiple culture conditions as demonstrated here. Since widefield ORI does not perform optical sectioning, signals are integrated over ∼100  μm, which may be advantageous for rapidly sampling multiple depths within 3D samples. Compared with single-cell segmentation and analysis, FOV-level analysis also greatly decreases computational time, which could enable real-time monitoring during biomanufacturing.

Intensity-based measurements such as ORI generally need to be normalized to account for day-to-day system variability and changes in background effects. Here, we note that our synthetic hydrogels are weakly autofluorescent in the DAPI and FITC channels, potentially due to both material properties and the sequestration of cell-secreted factors over time (Fig. 2). This background signal may shift over time and likely contributes a small amount to the overall signal. We accounted for daily system variations and background contribution by normalizing the cell autofluorescence to the fluorescence intensity of the cell-free gel (background) in both channels before calculating ORR. This normalization was performed on an individual gel level, accounting for any differences that may occur in focus or exposure between gels. For comparison, we also included ORR measured from cells on coated plates at each timepoint to assess the metabolism of cells on novel hydrogels relative to a biological standard. We note that a standard fluorophore (e.g., rhodamine) or day-matched cell-free gel could also be used to account for the background variations and contribution.

In this study, we seeded all gels with cells from the same initial cardiac progenitors so that metabolic changes can be attributed to differences in substrate composition. The protocol is expected to produce a relatively pure CM population^3^ and observed metabolic changes dominated by the differentiation process in iPSC-CMs^46^ rather than proliferation or senescence.^69^^–^^71^ We found that ORI can detect increased oxidative activity during early maturation (decreased ORR over time) and separates cells by differentiation efficiency. Furthermore, we demonstrated the ability to perform extended culture and repeated ORI on synthetic hydrogels. ORR continued to decrease beyond the early maturation (day 30) stages, indicating continued metabolic shifts over 100 days. We note that this trend is more robust in cells seeded on synthetic hydrogels [Fig. 5(b), magenta] relative to Matrigel or Geltrex (gray): significant decreases in ORR continue in synthetic gels alone from early to late maturation (Fig. S1 in the Supplementary Material). These differences may be due to known difficulties with extended culture on Matrigel, as it tends to degrade in an uncontrollable manner over time.^25^

The hydrogel conditions tested here were selected both for their relevance to the cardiac environment and their ability to support CPC adhesion and survival. This considerably limited the number of substrate conditions that were able to be tested in these experiments. This may have contributed to the lack of sensitivity of widefield ORI to substrate-induced metabolic changes. We found that widefield ORI is not sensitive to metabolic changes induced by substrate differences studied here (e.g., stiffness, concentration of primary and/or secondary peptide).

Currently, standard methods to determine differentiation efficiency in stem cell batches or maturation of the resulting cell types are labor-intensive and destructive, confining these techniques to a single timepoint. By contrast, our workflow is medium throughput and uses a standard commercial widefield system that is commonly found in biological laboratories and free-to-use software (ImageJ/Fiji), which could be widely adopted by academic and industrial groups that do not have significant optics experience. Future improvements to this workflow will include streamlining the background normalization steps to further reduce computational time. While widefield ORI cannot distinguish between specific metabolic pathways since it relies on whole cell NAD(P)H and FAD signals, the touch-free nature of this method enables repeated measurements at any point (stem cell culture through maturation) and can be combined with standard metabolic perturbations.^46^ Furthermore, widefield ORI was not found to affect iPSC-CM viability or purity (Fig. 3), and all flow cytometry was performed on imaged cells. This demonstrates that the imaged cells can later be used for complementary endpoint analyses in the future such as immunostaining, quantitative proteomics or metabolomics,^72^ and single-cell RNA sequencing.^73^^,^^74^

## 5 Conclusions

We demonstrated a label-free widefield imaging technique to monitor the metabolic phenotype of iPSC-CMs differentiating and maturing on synthetic hydrogels. This method is medium throughput and uses accessible microscope modalities and analysis tools. Multiphoton FLIM confirmed expected metabolic changes in iPSC-CMs during maturation on hydrogels. We demonstrated using widefield ORI that glycolytic metabolism decreases in differentiated cells during early and late maturation in iPSC-CMs cultured on synthetic hydrogels, and the same cells can be repeatedly monitored and used for complementary analyses. Furthermore, widefield ORI distinguishes between high and low differentiation efficiency batches in the early CPC stage. Overall, these tools provide an accessible, medium throughput method to monitor iPSC metabolism in response to interventions that aim to increase batch yield, consistency, and maturity without manipulating or destroying the cells.

## Supplementary Material

10.1117/1.BIOS.1.1.015002.s01

## Data Availability

Data and code to generate figures are available: https://github.com/skalalab/desa_d_hydrogel-widefield-ORI
