# Supplementary material for: Optical redox imaging to screen synthetic hydrogels for stem cell-derived cardiomyocyte differentiation and maturation
Source: Biophotonics Discov. 2024 May 20;1(1):015002. doi: 10.1117/1.BIOS.1.1.015002 (PMC11258857; doi:10.1117/1.BIOS.1.1.015002)
Supplement: Supplementary file 1 [file BIOS_001_015002_SD001.pdf]

## Supplementary Information

### Table S1. General synthetic gel information

| Peptide | Primary/secondary | Abbreviation | Derived from |
|---|---|---|---|
| Linear RGD (arginylglycylaspartic acid) | **1°** | L | Fibronectin |
| Cyclic RGD (arginylglycylaspartic acid) | **1°** | C | Fibronectin |
| IKVAV (Ile-Lys-Val-Ala-Val) | **2°** | I | Laminin |
| YIGSR (Tyr-Ile-Gly-Ser-Arg) | **2°** | Y | Laminin |

### Table S2. FLIM differentiation formulations (Fig. 2)

| Concentration (mM) | 1° | Concentration (mM) | 2° | Stiffness (kPa) |
|---|---|---|---|---|
| 7 | L | - | - | 0.5 |
| 7 | L | 1.5 | I | 0.5 |
| 3.5 | C | - | - | 0.5 |
| 3.5 | C | 1.5 | Y | 0.5 |
| 7 | C | 3 | I | 0.5 |
| 7 | C | 3 | Y | 0.5 |
| 3.5 | L | - | - | 2 |
| 7 | L | - | - | 2 |
| 7 | L | 1.5 | I | 2 |
| 3.5 | C | - | - | 2 |
| 7 | C | - | - | 2 |
| 7 | C | 3 | Y | 2 |
| 3.5 | L | - | - | 4 |
| 3.5 | L | 3 | Y | 4 |
| 3.5 | C | - | - | 4 |
| 3.5 | C | 3 | I | 4 |
| 3.5 | C | 3 | Y | 4 |
| 7 | C | 3 | Y | 4 |
| 7 | L | 1.5 | I | 6 |
| 7 | L | 3 | Y | 6 |
| 3.5 | C | 1.5 | Y | 6 |
| 3.5 | C | 3 | Y | 6 |
| 7 | C | 1.5 | I | 6 |

| | | | | |
|---|---|---|---|---|
| 7 | C | 3 | Y | 6 |
| 7 | C | - | - | 10 |
| 7 | C | 1.5 | I | 10 |
| 7 | C | 3 | I | 10 |
| Matrigel | | Gel format | | < 1[25] |
| Geltrex | | Gel format | | < 1[25] |
| Matrigel | | Coated plate | | - |

**Table S3. ORI differentiation formulations (Fig. 3-4)**

| Concentration (mM) | 1° | Concentration (mM) | 2° | Stiffness (kPa) |
|---|---|---|---|---|
| 7 | L | - | - | 2 |
| 3.5 | C | - | - | 2 |
| 7 | C | 3 | Y | 2 |
| 7 | L | 1.5 | I | 6 |
| 3.5 | C | 3 | Y | 6 |
| 7 | C | 1.5 | I | 6 |
| 7 | C | - | - | 10 |
| 7 | C | 1.5 | I | 10 |
| 7 | C | 3 | I | 10 |
| Matrigel | | Gel format | | < 1 |
| Geltrex | | Gel format | | < 1 |
| Matrigel | | Coated plate | | - |

**Table S4. Maturation formulations (Fig. 5A)**

| Concentration (mM) | 1° | Concentration (mM) | 2° | Stiffness (kPa) |
|---|---|---|---|---|
| 7 | L | 1.5 | I | 0.5 |
| 3.5 | C | - | - | 0.5 |
| 3.5 | C | 1.5 | Y | 0.5 |
| 7 | L | - | - | 2 |
| 7 | L | 1.5 | I | 2 |
| 3.5 | C | - | - | 2 |

| | | | | |
|---|---|---|---|---|
| 7 | C | 3 | Y | 2 |
| 3.5 | L | - | - | 4 |
| 3.5 | C | - | - | 4 |
| 3.5 | C | 3 | I | 4 |
| 7 | C | 3 | Y | 4 |
| 7 | L | 1.5 | I | 6 |
| 7 | L | 3 | Y | 6 |
| 3.5 | C | 3 | Y | 6 |
| 7 | C | 1.5 | I | 6 |
| 7 | C | - | - | 10 |
| 7 | C | 1.5 | I | 10 |
| 7 | C | 3 | I | 10 |
| Matrigel | | Gel format | | < 1 |
| Geltrex | | Gel format | | < 1 |
| Matrigel | | Coated plate | | - |

**Table S5. Maturation formulations (Fig. 5B)**

| **Concentration (mM)** | **1°** | **Concentration (mM)** | **2°** | **Stiffness (kPa)** |
|---|---|---|---|---|
| 3.5 | C | - | - | 0.5 |
| 3.5 | C | 3 | Y | 0.5 |
| 7 | L | - | - | 2 |
| 3.5 | C | - | - | 2 |
| 3.5 | C | 1.5 | Y | 2 |
| 7 | C | 3 | Y | 2 |
| 7 | C | 3 | I | 2 |
| 7 | L | 1.5 | I | 6 |
| 3.5 | C | 3 | Y | 6 |
| 7 | C | 1.5 | I | 6 |
| 7 | C | - | - | 10 |
| 7 | C | 1.5 | I | 10 |
| 7 | C | 3 | I | 10 |
| Matrigel | | Gel format | | < 1 |

| | | | | |
|---|---|---|---|---|
| Geltrex | | Gel format | | < 1 |
| Matrigel | | Coated plate | | - |

**Table S6. High vs. low formulations (Fig. 6)**

| Concentration (mM) | 1° | Concentration (mM) | 2° | Stiffness (kPa) |
|---|---|---|---|---|
| 7 | L | 1.5 | I | 0.5 |
| 3.5 | C | 1.5 | Y | 0.5 |
| 7 | L | - | - | 2 |
| 7 | C | 3 | Y | 2 |
| 3.5 | L | - | - | 4 |
| 3.5 | C | - | - | 4 |
| 3.5 | C | 3 | I | 4 |
| 7 | C | 3 | Y | 4 |
| 7 | L | 1.5 | I | 6 |
| 7 | L | 3 | Y | 6 |
| 3.5 | C | 3 | Y | 6 |
| 7 | C | 1.5 | I | 6 |
| 7 | C | - | - | 10 |
| 7 | C | 1.5 | I | 10 |
| 7 | C | 3 | I | 10 |
| Matrigel | | Gel format | | < 1 |
| Geltrex | | Gel format | | < 1 |

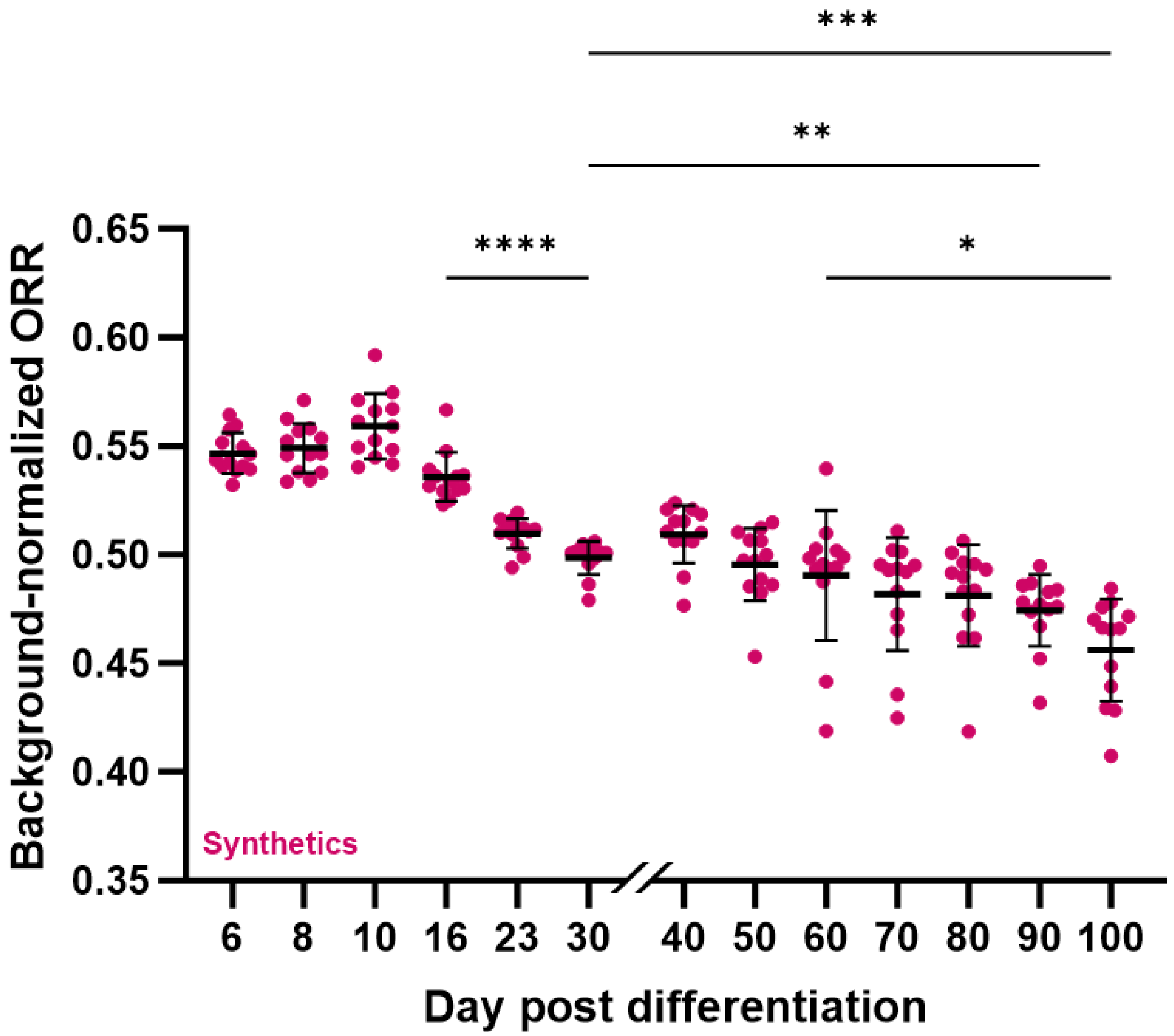


**Fig. S1. Widefield ORR becomes more oxidized in cells on synthetic hydrogels throughout late iPSC-CM maturation**. CPCs were seeded on synthetic hydrogels at the progenitor stage and repeatedly imaged through the differentiation (day 16), early maturation (day 30), and late maturation (>day 30) timepoints. ORR significantly declines beyond the early maturation timepoint, with continued decreases observed through day 100.
